# Do *Varroa destructor* (Acari: Varroidae) mite flows between *Apis mellifera* (Hymenoptera: Apidae) colonies bias colony infestation evaluation for resistance selection?

**DOI:** 10.1093/jisesa/ieae068

**Published:** 2024-07-11

**Authors:** Matthieu Guichard, Adrien von Virag, Benoît Droz, Benjamin Dainat

**Affiliations:** Agroscope, Swiss Bee Research Centre, Bern, Switzerland; Agroscope, Swiss Bee Research Centre, Bern, Switzerland; Agroscope, Swiss Bee Research Centre, Bern, Switzerland; Agroscope, Swiss Bee Research Centre, Bern, Switzerland

**Keywords:** bias, immigration, mite flow, resistance, selection

## Abstract

Since the global invasion of the ectoparasitic mite *Varroa destructor* (Anderson and Trueman), selection of mite-resistant honey bee (*Apis mellifera L.*) colonies appears challenging and has to date not broadly reduced colony mortality. The low published estimated heritability values for mite infestation levels could explain the limited genetic progresses obtained so far. We hypothesize that intercolonial horizontal mite transmission could differentially affect the single colonies located in a given apiary and therefore invisibly bias colony infestation phenotypes. This bias may be lower in regions with lower colony density, providing suitable conditions to set up evaluation apiaries. To verify these hypotheses, we monitored mite infestation and reinvasion in experimental colonies, as well as infestation in neighboring colonies belonging to beekeepers in three areas with variable colony densities in the canton of Bern, Switzerland during three consecutive beekeeping seasons. Mite immigration fluctuated between apiaries and years and significantly contributed to colony infestation level. Depending on apiary and year, 17–48% of the mites present in the experimental colonies at the time of the summer oxalic acid final treatment potentially derived from mite immigration that had occurred since mid-spring. Mite immigration was not linked to local colony density or the infestation levels of beekeepers’ colonies located within 2 km. Our results do not prove that apiaries for colony evaluation should necessarily be established in areas with low colony density. However, they highlight the high impact of beekeeping management practices on mite colony infestation levels.

## Graphical Abstract

**Graphical Abstract:**
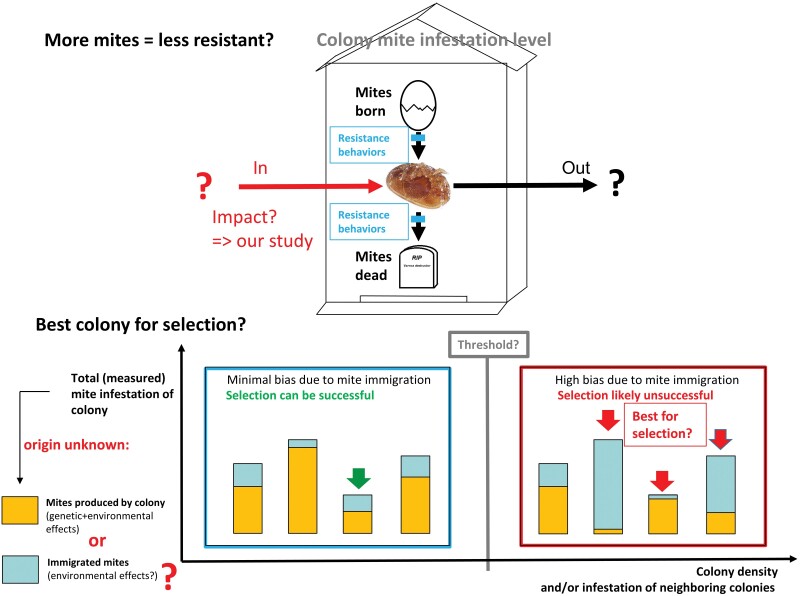

## Introduction

The ectoparasitic mite *Varroa destructor* (hereafter referred to as “mite”) is one of the main threats to the Western honey bee (*Apis mellifera*) (Rosenkranz et al. 2010, Roth et al. 2020, Traynor et al. 2020). *Varroa destructor* weakens honey bee brood and adults through feeding (de Jong et al. 1982, Bowen-Walker and Gunn 2001, Amdam et al. 2004, Ramsey et al. 2019, Han et al. 2024), and acts as a vector for several viruses, such as deformed wing virus (DWV) (Chen and Siede 2007, de Miranda and Genersch 2010, Genersch and Aubert 2010). The DWV-*V. destructor* combination is a reliable predictor of honey bee colony collapse (Carreck et al. 2010, Dainat et al. 2012a, 2012b, Barroso-Arévalo et al. 2019), as shown in numerous studies which documented the role of *V. destructor* in colony losses worldwide (e.g., Martin et al. 1998, Dahle 2010, Guzman-Novoa et al. 2010, Morawetz et al. 2019, Van Esch et al. 2019, Stahlmann-Brown et al. 2022).

Mite infestation level of managed honey bee colonies can be reduced by treatments with organic or synthetic miticides (Imdorf et al. 1996, Rosenkranz et al. 2010, Gregorc et al. 2016, Haber et al. 2019). However, such treatments are costly and are not always consistently implemented by beekeepers (Hernandez et al. 2022). In addition, they can contaminate bee products by leaving residues (Bogdanov 2006, Kast et al. 2019), have side effects on honey bees (Higes et al. 1999, Colin et al. 2020, Kast et al. 2023) and lead to the emergence of mite resistance (Elzen et al. 2000, Spreafico et al. 2001, Hernández-Rodríguez et al. 2021). Therefore, since the global invasion of *V. destructor*, alternative approaches favoring long-term colony survival, without the need for such treatments have been explored (Dietemann et al. 2012). These approaches include the selection of honey bees resistant to *V. destructor* (Harbo and Harris 1999, Büchler et al. 2010, Rinderer et al. 2010, Kefuss et al. 2016, Guichard et al. 2020a), that is, capable of keeping colony infestation at a bearable level, by means of defense mechanisms. Among the latter, suppressed mite reproduction (Harbo and Harris 2002, Buchegger et al. 2018, von Virag et al. 2022) and recapping of infested cells (Buchegger et al. 2018, Martin et al. 2019, Guichard et al. 2023) can decrease the quantity of mites which are born in the colony. Others, such as Varroa sensitive hygiene (Harbo and Harris 2009, Danka et al. 2016), where infested pupae are removed, and grooming (Fries et al. 1996, Morfin et al. 2020) can increase the number of mites which die. In previous studies selection targets were either colonies with low infestation or colonies with highly expressed defense mechanisms, in which lower infestation levels are expected (Guichard et al. 2020a).

Unfortunately, selection of mite-resistant colonies has not yet resulted in broad populations that can be kept without miticides (Guichard et al. 2020a). Thus far, success stories where mostly susceptible populations could be selected until becoming resistant and surviving are at best rare, and their long-term capacity to sustainably cope with their parasite is unknown. One main reason for the lack of breeding progress could be that, besides the honey bee genome, many environmental factors affect colony mite infestation level. These factors include location, climate, and beekeeping management practices, which account for a large part of the observed variation in mite infestation level (Garcia-Fernandez et al. 1995, Giacobino et al. 2017, Guichard et al. 2020a). Environmental effects that identically affect all colonies located in the same apiary or that can be estimated for each colony can be corrected for as fixed effects during breeding value and heritability estimation (Brascamp and Bijma 2014) and are not problematic for selection. In contrast, factors differently affecting individuals kept together in the same manner (here, honey bee colonies in an evaluation apiary) without being noticeable for the evaluator are likely to have a particularly negative impact on heritability estimates and estimated breeding values. Such factors cannot be accounted for as fixed effects (e.g., a single apiary effect) when estimating heritabilities and breeding values. These factors and their possible, yet largely unknown interactions, likely explain the very low estimated heritabilities of mite infestation level in several populations, with values often being below 0.10 and close to zero in studies on large populations (Guichard et al. 2020b, Hoppe et al. 2020, Bernstein et al. 2023). Under these conditions, variation in colony infestation levels can hardly be explained by genetic factors of the honey bees, making it challenging to select genetically superior colonies containing fewer mites.

Horizontal mite flows between honey bee colonies, occurring through drift and robbing (Goodwin et al. 2006, Peck and Seeley 2019, Kulhanek et al. 2021), may affect colonies in the same apiary differently, resulting in different impacts on their infestation levels. Mite flows between colonies may depend on many factors, which cannot be precisely predicted. For instance, foragers from one colony may by chance discover a collapsing colony in their flight radius, rob its honey stores, vector mites back and heavily increase the infestation level of their colony, whereas workers from a neighboring colony may forage in another direction and therefore not import the same number of mites. As an analogy, different foraging patterns among colonies lead to very different floral origins of harvested pollen, even if the colonies are placed side by side (Roncoroni et al. 2021).

The main assumption underlaying colony evaluation in a selection program -including for lower mite infestation levels- is that all colonies in the same apiary share identical environmental conditions (Büchler et al. 2013, Uzunov et al. 2017), enabling interpretation of phenotypic variation as caused by genotypic variation, which can then be exploited by selection. Such interpretation is not possible if every colony shapes an individual network of mite flows with its neighbors. Ultimately, the more horizontal mite flows vary between single colonies because of unpredictable events, the less the mite infestation level can be related to the genome of the colony and to resistance traits, hindering estimation of reliable heritabilities and breeding values.

Horizontal mite flows or so-called “reinvasion” have been shown to occur at a greater extent in regions with a high colony density (Ritter and Leclercq 1987, Frey and Rosenkranz 2014). More interactions probably occur among colonies in regions with high colony densities, leading to greater opportunities for mite transfer from one colony to another. Under specific environmental conditions and depending on the season, up to several hundred mites can enter honey bee colonies per day (Imdorf and Kilchenmann 1991). Even at lower magnitudes, incoming mite flows can increase the infestation level of the recipient colonies (Ritter and Leclercq 1987, Frey and Rosenkranz 2014). In Europe, mite flows mostly occur in the fall (Sakofski et al. 1990, Büchler and Hoffmann 1991, Greatti et al. 1992) but can also take place in summer (Ritter and Leclercq 1987, Greatti et al. 1992) and, at a lower extent, in spring (Sakofski et al. 1990). As honey bee selection programs generally evaluate mite infestation levels between early spring and application of summer miticide treatments (Büchler et al. 2013), mite immigration during this period could therefore bias the recorded mite infestation levels. Horizontal mite flows can span several kilometers (Frey et al. 2011) without being detectable for the beekeeper, making the outcome of infestation evaluations depend not only on beekeeping practices in each apiary where phenotypes are recorded, but also on those of neighboring beekeepers.

The aim of this study was to investigate whether biases in measured infestation levels due to horizontal mite flows between colonies can be estimated and whether such biases can be anticipated based on knowledge of the local colony density and the infestation levels of neighboring colonies. If this was possible, recommendations could be made to breeding organizations regarding maximum local colony density thresholds when establishing apiaries for colony evaluation. An accurate location choice for the setup of apiaries could then lead to less biased infestation levels, possibly higher estimated heritabilities and more reliable estimated breeding values, enabling successful selection of *V. destructor*-resistant colonies.

## Materials and Methods

### Study Area

**Fig. 1.**
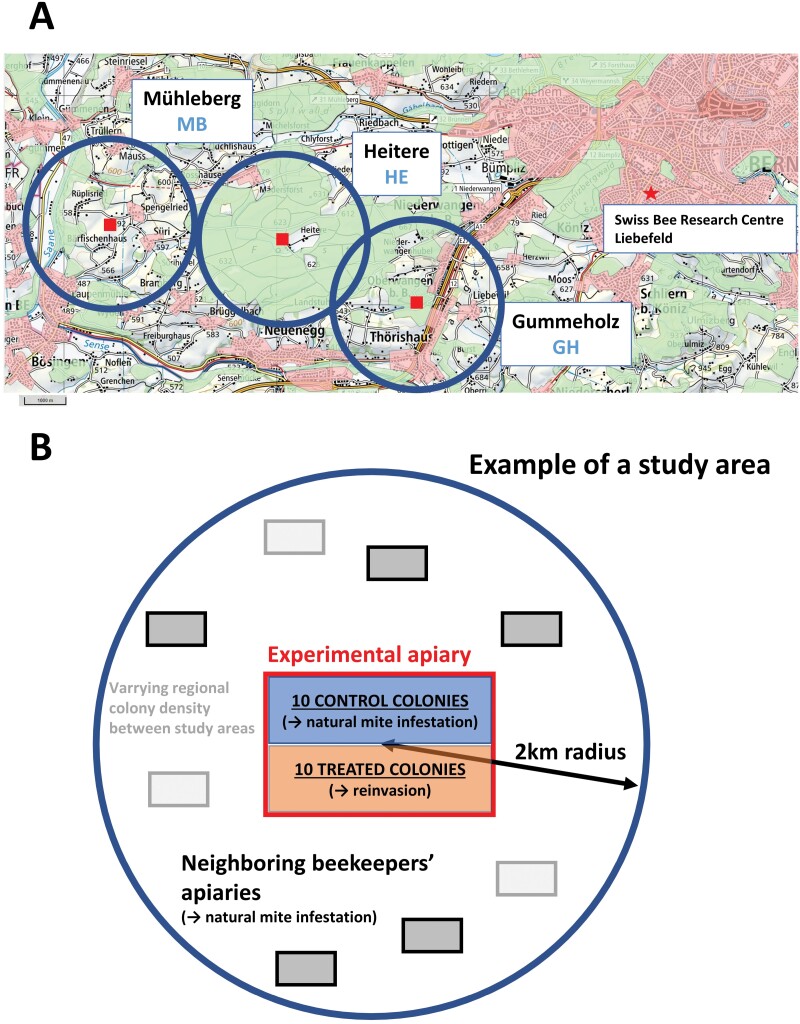
A) Map of the three study areas. Each experimental apiary (red square) and the 2-km radius surrounding the study area (blue circle) are represented. The overlapping area between the two eastern zones (HE and GH) contained no apiaries. Landcover data from the swissTLMRregio map provides polygons with land use information: orange = built-up area and inhabited zone, green = forest, area without polygons: agricultural area. Map background and swissTLMRregio map *©* Swisstopo, reproduced with permission. B) Graphical representation of a 2-km radius study area with the experimental apiary in its center and a variable number of apiaries of neighboring beekeepers (gray tones).

The study was performed between late April and mid-August 2021, 2022, and 2023 in the canton of Bern, Switzerland. Three study areas, Mühleberg (MB), Heitere (HE), and Gummeholz (GH), close to the city of Bern (Fig. 1A) were selected with the help of the cantonal veterinary services. The three study areas fulfilled the following requirements: varying colony densities, estimated through the number of yearly registered colonies, similar environmental conditions, among which altitude around 600 m above sea level in a forest and agriculture landscape; and ease of access from the Swiss Bee Research Centre in Bern.

Each study area was a circle with a radius of 2 km, chosen to roughly correspond to the immediate flight range of honey bees in Europe (Couvillon et al. 2014, Danner et al. 2016, Rutschmann et al. 2023), and therefore covered an area of 12.6 km^2^. The land use type in each of the three study areas is shown in Fig. 1A.

### Experimental Apiaries

In the center of each study area, an experimental apiary of 20 *Apis mellifera mellifera* colonies was established (Fig. 1B). To study environmental effects, such as incoming mite flows, all colonies of a given year were headed by tagged sister queens. These queens were mated to drones descending from sister queens (i.e., one single paternal origin per year) at a mountain mating station managed by the breeding organization mellifera.ch (www.mellifera.ch). Each year, the queens for the following experimental season were reared from a single colony chosen for its satisfying abilities for beekeeping, namely: bees covered at least 10 Dadant frames and exhibited moderate defensive behavior.

In 2021, all colonies were started from nuclei on three brood frames (Büchler et al. 2013). However, the colonies exhibited a high swarming behavior, accompanied by queen loss and requeening, with possibly high impacts on mite infestation levels, leading to nonexploitable data, removed from the analysis. To solve this problem, in 2022 and 2023, the colonies were started from 1.5 kg honey bee packages placed on worker-sized wax foundations (Büchler et al. 2013). These colonies did not exhibit swarming behavior. In 2022, the 60 colonies were successfully maintained with their original queen until the end of the experiment. In 2023, three colonies out of 60 lost their queens. The latter colonies received a replacement queen and were kept at their original location so as to maintain a constant number of colonies in each apiary and minimally impact between-colony mite flows. However, the data from these three colonies were not included in the analysis, as queen replacement could have affected their mite infestation dynamics.

The experimental colonies were enlarged with additional foundations, fed and equipped with a honey super when necessary, according to Swiss beekeeping practice. Colony size was evaluated every three weeks using the Liebefeld method (Imdorf et al. 1987, Dainat et al. 2020) (cf. dedicated analysis as Supplementary Material SM1). Between consecutive years of the experiment, the experimental colonies did not receive any winter miticide treatment, to ensure that the infestation levels of the colonies would be high enough to enable mite counts the following spring.

The colonies (*n* = 20) in each experimental apiary were split into two groups (Fig. 1B):

- One group (*n *= 10 colonies) was maintained under standard beekeeping conditions (control group). In this group, the undisturbed mite infestation level was monitored by continuous natural mite fall records during each experimental season. For standardization of mite infestation levels of the colonies in the control group in each experimental apiary at the start of the study, the bees from the different packages were gathered in a large box, left to homogenize, and later separated again to form the experimental colonies.
- The other group (*n* = 10 colonies) received a continuous miticide treatment (treated group) allowing to attribute the recorded mite fall to mite immigration by suppressing mite reproduction in the colony. Treatment started five days after colony establishment, combining coumaphos (CheckMite®; Bayer Healthcare AG) and flumethrin (Bayvarol®; Bayer Healthcare AG). The protocol was inspired by Frey and Rosenkranz (2014) and slightly modified: each colony received one strip of CheckMite® combined with one strip of Bayvarol® to minimize the risk of bias due to mite resistance to one of the compounds (Frey and Rosenkranz 2014), and the strips were replaced after eight weeks to guarantee optimal efficacy. Monitoring of incoming mite flows through mite counts on the bottom boards was initiated 21 days after the initial treatment application in order to start the experiment with virtually mite-free colonies.

The control and treated groups were separated by a few meters within each apiary to minimize intergroup transfer of miticides. We did not homogenize the between-group mite infestation level before the start of the experiment, as the same stock was used from year to year to produce the experimental colonies, and we did not want to contaminate the control colonies of one year with the two lipophilic miticides through bees originating from colonies treated in the previous year.

### Mite Infestation Estimates

We obtained four separate estimates of mite infestation on the control and treated colonies. These include the sum of natural mite fall, adult worker infestation in spring, adult worker infestation in summer and final colony infestation level. We describe each estimation method below.

In both groups, adult mite fall was evaluated continuously between spring and summer (from May 17 to July 12 in 2022 and May 17 to July 13 in 2023) by examining the bottom boards of the hives every two to four days and counting the dead mites present according to standard protocols (Büchler et al. 2013, Dietemann et al. 2013). To avoid mite collection by ants and subsequent biases in data (Dainat et al. 2011), the boards were covered with an absorbent paper soaked with plant oil (Dietemann et al. 2013), which was replaced following every evaluation. The colonies were also placed on elevated stands with arboricultural glue coating the pillars to prevent ant access (Dietemann et al. 2013). In both groups, the number of fallen mites over each sampling time range was divided by the number of days since the last count to obtain the number of mites per day. The evolution of daily mite fall was plotted as a function of time. The sum of natural mite fall for each colony during the experiment was also calculated.

Mite infestation level of workers in spring and summer, was obtained by the washing method (Dietemann et al. 2013). For this, samples of 300 adult workers were taken from all experimental colonies at the beginning (May 19 in 2022 and May 17 in 2023) and the end (July 12 in 2022 and July 13 in 2023) of the experimental seasons and later processed following standard protocols (Dietemann et al. 2013).

The final colony infestation level was obtained as follow for both the control and the treated groups: at the end of the experimental seasons, the queens were caged for 25–26 days (July 17 to August 05 in 2022 and July 13 to August 07 in 2023). The broodless colonies then received a final miticide treatment on August 05 in 2022 and August 07 in 2023. Three to four milliliters of 3% oxalic acid solution (Oxuvar® 5.7%; Andermatt BioVet AG) were sprayed on each comb side occupied by bees. Given the very high efficacy of oxalic acid against *V. destructor* (Radetzki 1994, Rademacher and Harz 2006, Rosenkranz et al. 2010), mite fall evaluated on the bottom boards during the 2 weeks following the final summer oxalic acid treatment (August 05 to August 19 in 2022 and August 07 to August 21 in 2023) was interpreted as the final colony infestation level.

After pooling the data from 2022 and 2023, the pairwise correlations coefficients (Kendall’s tau) between the four estimates of colony infestation level (i.e., sum of natural mite fall, adult worker infestation in spring, adult worker infestation in summer, and final colony infestation estimated by mite fall after the final oxalic acid treatment in summer) retrieved from the control colonies were calculated in R (R Core Team 2018).

In the treated group, we hypothesized that every counted mite had been killed by the continuous miticide treatment immediately after entering the hive and before it could enter a brood cell to reproduce (Frey and Rosenkranz 2014). Treatment efficacy was verified using three complementary validation methods in 2022 and 2023:

- The above-mentioned washed worker samples: the treated colonies were expected to contain nearly zero mites if they had been mite free during the whole experiment.
- An evaluation of the number of light-colored juvenile mites found on the bottom boards: the treated colonies were expected to have nearly no light-colored mites on the boards, as such mites would indicate that mite reproduction had taken place.
- The final colony infestation level: the treated colonies were expected to have a very low final infestation, estimated from the final oxalic acid treatment mite fall.

### Impact of Mite Immigration

In a following step, the potential impact of mite immigration on the treated colonies was estimated. The general idea behind this analysis, detailed hereafter, was to consider a virtual situation where the colonies of the treated group would have been kept untreated during the experiment. For this, we hypothesized that, if the continuous miticide treatment had not been applied throughout the experimental period, each founder mite would have completed two successive reproduction cycles (Fries and Rosenkranz 1996, Martin and Kemp 1997) and would have produced on average 1.1 mature female offspring per cycle, based on recent observations on the same *A. m. mellifera* population (von Virag et al. 2022). Given that we provided only worker-sized wax foundations, the colonies only contained very limited amounts of drone brood, which we neglected in our estimation of mite reproduction. To simplify calculations, we assumed that the duration of the mite’s reproductive cycle was the same as that of the worker brood (21 days), a situation that has been observed in the field (Fries et al. 1994, Beetsma et al. 1999). This duration however could vary and be shorter in practice, depending on the length of the mobile phase on the adult bees and the ages of the mites (Fries et al. 1994, Beetsma et al. 1999). Our rather conservative evaluation enabled us to estimate, for each treated colony, the minimum theoretical number of mites at the end of the experimental season that could be attributed to mite immigration if the colonies of the treated group had not received any continuous miticide treatment.

By subtracting the effect of mite immigration (obtained from the treated group) from the final colony infestation level (obtained from the control group), we could later estimate unbiased infestation levels for the control colonies of each experimental apiary in absence of any immigration. This extrapolation was enabled by the fact that all queens were sisters. Therefore, we expected that the queens of the control and treated groups had equivalent average genetic values. The possible range of unbiased infestation levels obtained in this manner was plotted for each control colony of each apiary. Using a Kruskal–Wallis rank-sum test followed by post-hoc pairwise Wilcoxon rank-sum tests (*P-*value adjustment method of Benjamini–Hochberg) in R (R Core Team 2018), we tested whether unbiased infestation levels differed significantly between colonies of the same apiary. Significant differences would mean that the colonies could clearly be distinguished based on their infestation level, enabling secure choices for selection. In contrast, nonsignificant differences would indicate that the visible variation in infestation levels would likely be too low to be exploited by selection. The proportions of pairwise comparisons leading to significant differences were compared between the experimental apiaries, and provide the main result of our experiment.

### Colonies of Neighboring Beekeepers

To identify possible mite sources that could have caused immigration into the experimental colonies, beekeepers with registered apiaries located in each study area, within 2 km of each experimental apiary (Fig. 1B) were subsidized (fixed amount per year of participation plus amount for each taken sample, budgeted for in the research project) to take samples of 300 adult workers from each colony headed by a laying queen, excluding mating nuclei and Miniplus colonies on a single element, twice a year. We did not document nonregistered apiaries or feral colonies that could have been added to the sampling within the study areas. Spring sampling took place within a 16-day period in late April, and summer sampling took place within an identical time window around mid-July. As not all the beekeepers kept marked queens or were able or willing to find their queens during sampling, they were advised to take the samples from a lateral frame (pollen/honey stores) or from the honey super, where the queen was less likely to be present, as recommended by a recent field protocol (Uzunov et al. 2021). This method was preferred to the more usual sampling method involving brood frames (Lee et al. 2010) to avoid queen losses in the beekeepers’ colonies, which could have restricted their willingness to participate in the study. The number of workers per sample was set to 300, less than the recommended three times 300 workers per colony for high precision sampling (Lee et al. 2010), to favor the participation of beekeepers, often reluctant to kill bees. To enable comparisons, we used the same size of samples for the colonies of the experimental apiaries. To limit experimental bias, the beekeepers did not receive any recommendations on how to manage their colonies. The only information they were given was the measured mite infestation level of their colonies at the end of each beekeeping season. The beekeepers may have sampled the same colonies across years and seasons. They may also have imported or exported colonies from the study areas, split colonies, created nuclei, or experienced colony losses between sampling dates. Due to the absence of information on these beekeeper practices, the samples were not considered as repeated measures.

The samples were washed with soapy water at the Swiss Bee Research Centre using a standard protocol (Dietemann et al. 2013) to estimate colony infestation level. Infestation levels were represented per apiary, season, and year, with a mention of the number and proportion of samples exceeding the local empirical maximum infestation level thresholds (one mite per 100 workers in spring and four mites per 100 workers in summer). A generalized linear model (GLM) was run in R (R Core Team 2018) to evaluate the effects of study area, year, season, apiary (nested in the study area) and the interaction of the study area and year on mite infestation level of these neighboring colonies.

## Results

### Validation of Mite Immigration Monitoring

**Fig. 2.**
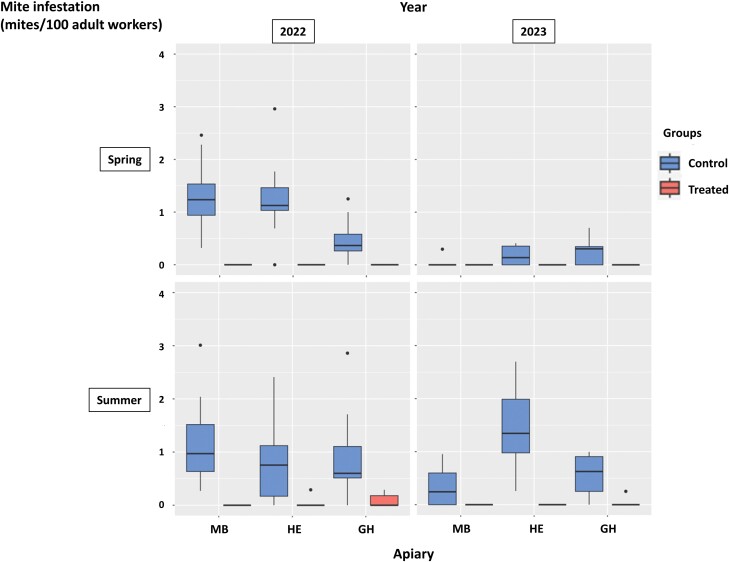
Mite infestation level (mites/100 adult workers) estimated by washing worker samples obtained from the colonies in the three experimental apiaries MB, HE, and GH in 2022 and 2023. Data are represented according to group (control or treated). The box plots represent the minimum value and the first quartile, median, third quartile, and maximum values. The dots indicate points located more than 1.5 times above or below the interquartile range.

**Fig. 3.**
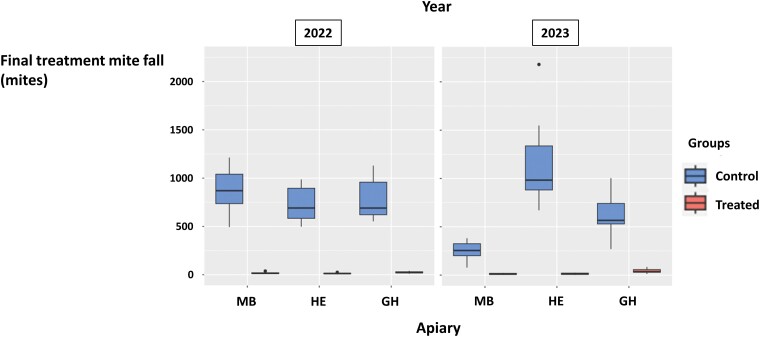
Number of fallen mites following the final oxalic acid treatment of the colonies in the three experimental apiaries MB, HE, and GH in 2022 and 2023. Data are represented according to group (control or treated). The box plots represent the minimum value and the first quartile, median, third quartile, and maximum values. The dots indicate points located more than 1.5 times above or below the interquartile range.

The infestation levels obtained from the adult workers sampled in the experimental colonies that kept their original queen until the end of the experiment are presented in Fig. 2. Overall, 296 mites were collected in the samples obtained from the control colonies (*n* = 58) in spring and summer, whereas only 5 mites were found in the samples from the treated colonies (*n* = 59), all of which were found in the summer samples. The mites in the treated group represented 1.7% of the mites in the control group. Following mite counts on the bottom boards during the experiment (Supplementary Material SM2), an average of 33.4 and 0.4 light-colored juvenile mites were found per colony in the control and treated groups, respectively. Juvenile mites in the treated group represented 1.2% of the ones in the control group. The final oxalic acid summer treatment dislodged, on average, 746 and 21 mites in the control and treated groups, respectively. Mites in the treated group represented 2.8% of those in the control group (Fig. 3). Despite practical constraints not having allowed mite infestation levels to be initially equalized between groups, the three validation methods yielded similar results, suggesting a very high efficacy of the continuous miticide treatment in all the experimental apiaries. Therefore, the hypothesis that the mites counted on the bottom boards of the treated groups were the result of mite immigration was validated.

### Mite Infestation, Immigration, and Colony Discrimination in the Experimental Apiaries

**Fig. 4.**
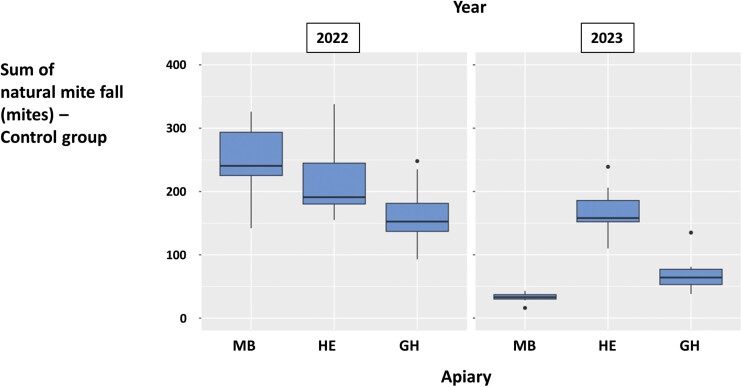
Sum of natural mite fall of the control colonies from the three experimental apiaries MB, HE, and GH in 2022 and 2023. The box plots represent the minimum value and the first quartile, median, third quartile, and maximum values. Dots indicate points located more than 1.5 times above or below the interquartile range.

**Fig. 5.**
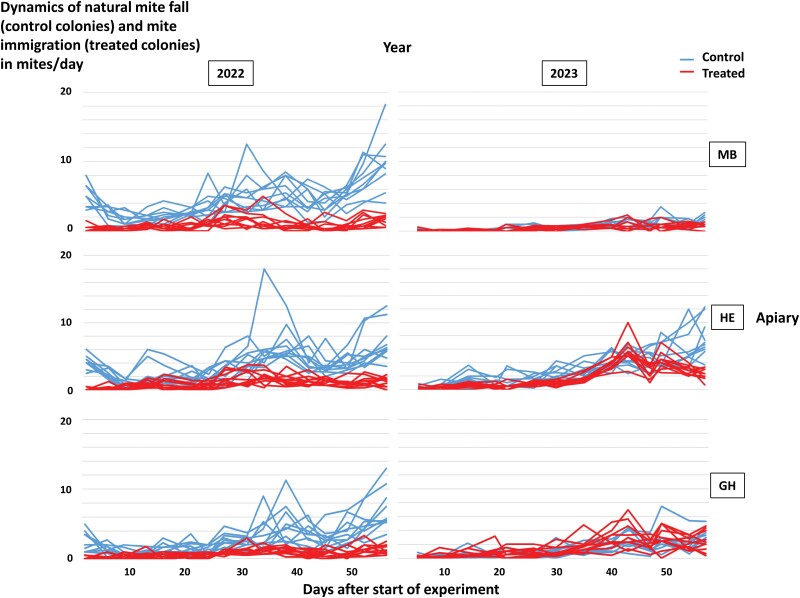
Dynamics (mites/day) of natural mite fall (control colonies, blue lines) and mite immigration (treated colonies, red lines) in number of mites per day during the experiment. Each line represents a colony. Data are represented according to year (2022 and 2023) and apiary (MB, HE, GH).

In the control colonies, the initial mite infestation level based on the spring adult worker samples (Fig. 2) was higher in 2022 than in 2023. In addition, more mites were counted during natural mite fall monitoring in 2022 than in 2023 (Fig. 4). In 2022, a peak in daily natural mite fall was observed in all apiaries around 30–40 days after the start of the experiment. Infestation levels also increased after the 50th day of the experiment (Fig. 5). In 2023, daily natural mite fall values showed a more constant increasing tendency over time (Fig. 5). Summer mite infestation level based on the analysis of the washed samples of adult workers was between 0 and 3 mites/100 bees in both years, although a tendency to greater variability was observed between the three experimental apiaries in 2023 than in 2022 (Fig. 2). The four estimates of colony infestation level (sum of natural mite fall, adult worker infestation in spring, adult worker infestation in summer, and final colony infestation estimated by mite fall after the oxalic acid treatment in summer) showed low-to-moderate correlations with each other in 2022 and 2023. The sum of natural mite fall had the highest correlation (τ = 0.54) with the final colony infestation level (Table 1).

**Table 1.** Pairwise correlations between the four mite infestation estimates obtained for the control colonies (adult worker infestation in spring, adult worker infestation in summer, final mite infestation estimated by the summer oxalic acid treatment mite fall and sum of natural mite fall) for 2022 and 2023 (*N* = 58). Kendall’s tau b coefficients (τ) are given, as well as *P*-values (*P*) associated with each correlation

	Sum of natural mite fall	Adult worker infestation in spring	Adult worker infestation in summer
Adult worker infestation in spring	τ = 0.51, *P* = 6.1e^−8^		
Adult worker infestation in summer	τ = 0.24, *P* = 7.6 e^−3^	τ = 0.22, *P* = .02	
Final mite infestation (summer oxalic acid treatment mite fall)	τ = 0.54, *P* = 3.0e^−^9	τ = 0.24, *P* = .01	τ = 0.28, *P* = 2.1e^−3^

**Fig. 6.**
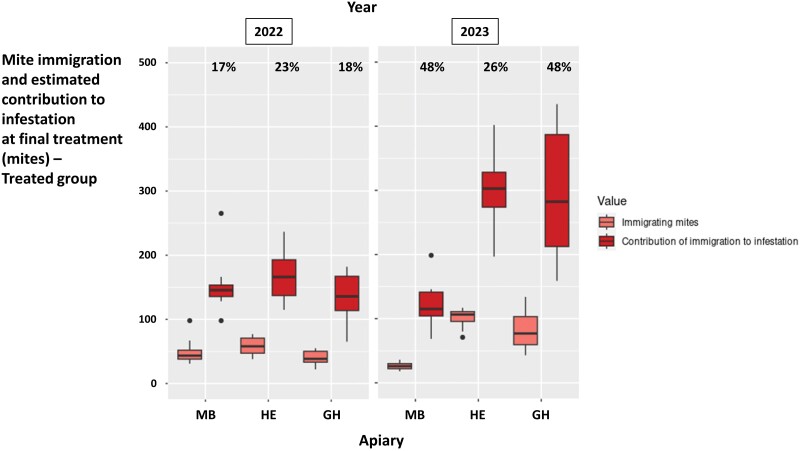
Number of immigrating mites of the treated colonies from the three experimental apiaries MB, HE, and GH in 2022 and 2023 and their estimated contribution to final colony infestation level. The box plots represent the minimum value, first quartile, median, third quartile, and maximum values. The dots indicate points located more than 1.5 times above or below the interquartile range. The percentages in the upper part indicate the proportion of mites in the control colonies at the time of the final oxalic acid treatment that could be attributed to mite immigration.

In the treated colonies, higher mite immigration was found in 2023 compared to 2022 in two of the three apiaries (Fig. 6). In addition, the dynamics of mite immigration varied between years and apiaries. In 2022, mite immigration was moderately elevated between days 30 and 40, before decreasing (Fig. 5). In 2023, the values remained low until day 30, when two peaks occurred around days 40 and 50, especially in the apiaries HE and GH (Fig. 5). The impact of mite immigration on final colony infestation level was highest in the apiaries HE and GH in 2023 (around 300 mites per colony), while the results obtained in the other experimental colonies were more similar (around 100 to 200 mites per colony) (Fig. 6).

The possible impact of mite immigration on final infestation level in the control colonies, estimated from immigration in the treated colonies, is presented in Fig. 6. The highest mean impact of mite immigration on the final colony infestation level and the highest variation of this impact among colonies of a single apiary were estimated for the HE and GH apiaries in 2023, respectively (Fig. 6 and Supplementary Material SM3). Overall, 19% and 41% of the final colony infestation level could be attributed to mite immigration in 2022 and 2023, respectively.

**Fig. 7.**
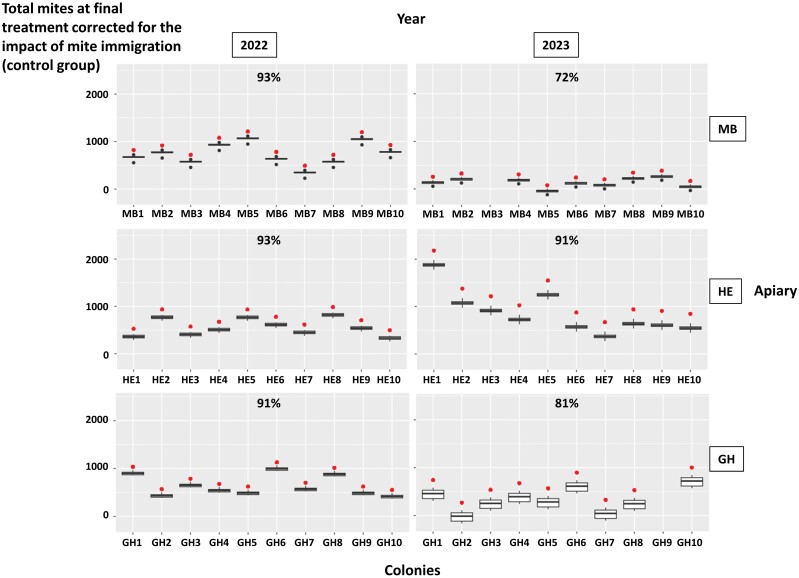
Total mites at the time of the final oxalic acid treatment in the control colonies corrected for the impact of mite immigration (estimated based on the treated colonies from the same apiary) for the three experimental apiaries in MB, HE, and GH in 2022 and 2023. The top red dots indicate the measured final colony infestation levels. The box plots indicate the distribution of the final colony infestation levels corrected for the impact of mite immigration estimated on the treated colonies located in the same apiary. The box plots represent the minimum value and the first quartile, median, third quartile, and maximum values. The black dots indicate points located more than 1.5 times above or below the interquartile range. Data from colonies that did not keep their original queen until the end of the experiment are not represented in the graph. The percentages in the upper part indicate the proportion of pairwise between-colony comparisons with significant differences between colonies following pairwise Wilcoxon rank-sum tests.

The influence of mite immigration on the choice of colonies for selection based on their final infestation level is presented in Fig. 7 and Supplementary Material SM4. In 2022, depending on the experimental apiary, 91–93% of the pairwise comparisons between colonies in the same apiary revealed significant differences between their final infestation levels corrected for the impact of mite immigration. In 2023, this proportion dropped to 72% in the apiary MB and 81% in the apiary GH.

### Infestation of Colonies Kept by Neighboring Beekeepers

The overview of the samples of worker bees that could be obtained between 2021 and 2023 from the neighboring colonies, located within 2 km of the experimental apiaries, is available in Table 2. Overall, more than 80% of the registered colonies located in the target area could be sampled. The remaining beekeepers did not wish to take samples themselves or to provide access to their apiaries. The range of colony densities in the study areas varied between 2.3 and 10.0 colonies per km^2^ (Fig. 1A, Table 2). Depending on the year, 79–84% of the registered apiaries provided samples. The colony density (excluding the colonies of the experimental apiaries) varied during the course of the experiment. In 2021 and 2022, the highest colony density (10 colonies/km^2^) was in the study area of GH, whereas the highest colony density in 2023 was in the study area of MB (9.6 colonies/km^2^). The lowest colony density, varying between 2.3 and 2.5 colonies/km^2^, was always recorded in the study area of HE.

**Table 2.** Number of apiaries, colonies, and samples from the neighboring beekeepers in the three study areas for 2021–2023. Totals per year and percentage of apiaries and colonies included in the study appear in italics. The numbers of apiaries and colonies were provided by the veterinary services of Canton Bern and are based on beekeeper’s declarations. Apiaries with zero registered colony were excluded. The number of samples can differ from the number of colonies of the participating registered apiaries, as registration of apiaries and colonies takes place on a yearly basis

Year	Study area	Registered apiaries (among which participating)	Registered colonies (among which from participating apiaries)	Density of registered colonies (col/km^2^)	Samples spring	Samples summer
2021	MB	15 (12)	104 (85)	8.3	88	93
	HE	3 (3)	29 (29)	2.3	27	28
	GH	19 (16)	126 (96)	10.0	89	117
	*Total*	*37 (31)*	*259 (210)*		*204*	*238*
	*% included*	*84%*	*81%*			
2022	MB	15 (12)	109 (99)	8.7	96	115
	HE	4 (3)	29 (29)	2.3	27	28
	GH	19 (15)	126 (93)	10.0	58	98
	*Total*	*38 (30)*	*264 (221)*		*181*	*241*
	*% included*	*79%*	*84%*			
2023	MB	15 (13)	120 (111)	9.6	89	107
	HE	4 (3)	31 (27)	2.5	28	31
	GH	19 (14)	85 (52)	6.8	92	112
	*Total*	*38 (30)*	*236 (190)*		*209*	*250*
	*% included*	*79%*	*81%*			

**Fig. 8.**
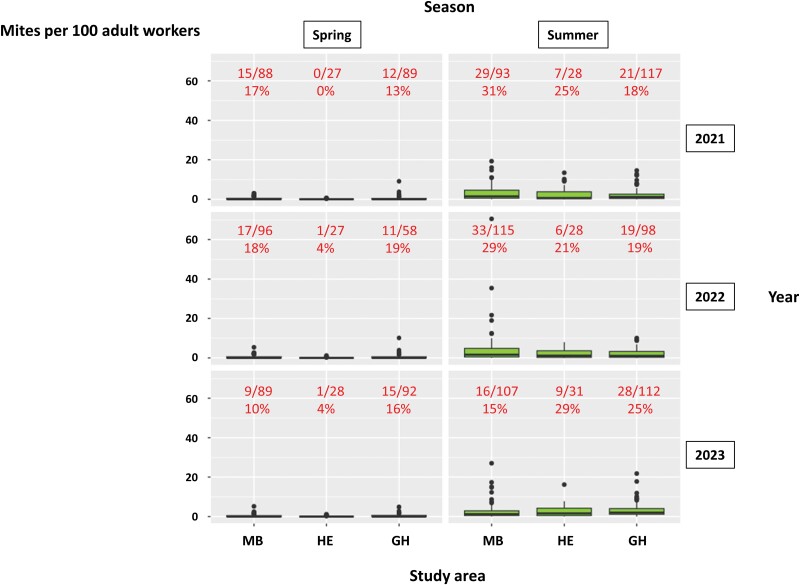
Infestation level (mites/100 adult workers) in samples taken from the neighboring beekeepers’ colonies located within each study area (MB, HE, and GH) in 2021, 2022, and 2023. The box plots represent the minimum value and the first quartile, median, third quartile, and maximum values. The dots indicate points located more than 1.5 times above or below the interquartile range. The top red figures indicate the number and proportion of colonies with infestation levels above commonly recommended thresholds in Switzerland (1 mite/100 workers in spring, 4 mites/100 workers in summer).

The infestation levels of the samples are shown in Fig. 8, and details on these values are provided in Supplementary Material SM5. The mite infestation levels in spring varied between 0 and 10.11 mites/100 adult workers (median = 0, mean = 0.40), whereas the summer values varied between 0 and 70.55 mites/100 adult workers (median = 1.37, mean = 2.81). The proportion of samples with infestation levels exceeding the empirical local thresholds ranged between 0 and 31%, depending on the year and location, and was always higher in summer than in spring. The highest proportions were found in the MB area in the summers of 2021 (31%) and 2022 (29%) and in the HE area in the summer of 2023 (29%). The GLM analysis revealed that season, apiary and the study area*year interaction had a strong significant (*P-*value < 0.05) impact on mite infestation level but that study area and year considered separately did not (Table 3).

**Table 3.** GLM analysis for the mite infestation level of the adult worker samples collected from the beekeeper’s colonies located within the three study areas in spring and summer 2021–2023. *P*-values below .05 are indicated in bold. Test of the whole model: *F* value: 9.058 on 42 and 1280 DF, *P*-value: < 2.2e-16

Response variable	Source of variation	df	*F* value	*P*-value
Mite infestation of samples (mites per 100 adult workers)	Study area	2	2.48	0.08
	Year	2	1.08	0.34
	Season	1	193.62	**<2e-16**
	Study area: Apiary	33	5.02	**<2e-16**
	Study area*Year	4	3.53	**7.1e-3**
	Residuals	1280		
	Total	1322		

## Discussion

The present study aimed to estimate the impact of horizontal mite flows on colony infestation level during spring and summer, the period when colonies are usually evaluated in selection programs (Büchler et al. 2013), as well as the resulting biases during the subsequent selection process. The range of colony densities in our study areas was representative of beekeeping conditions in Switzerland (Charrière et al. 2018, von Büren et al. 2019). We validated a method to follow mite immigration and found that mite immigration between spring and summer clearly contributed to the final colony infestation level. However, the possibility to distinguish the colonies according to their unbiased infestation values was only moderately affected. Mite immigration was not linked to local colony density, and thus the accuracy of a given apiary for reliable colony selection could not be predicted based on local colony density.

The method of continuous acaricide treatment (coumaphos + flumethrin) for the recording of mite immigration (Frey and Rosenkranz 2014) proved its reliability. Almost no mites reproduced in the treated colonies (Supplementary Material SM2), and the latter had very few remaining mites at the end of the experimental seasons (Fig. 2).

Mite infestation dynamics clearly differed between 2022 and 2023 in the control colonies (Figs. 2–5). Surprisingly, infestation levels measured in spring had no direct impact on those recorded in summer: for instance, based on the washed adult worker samples, colonies in the experimental apiary HE had more mites in spring 2022 than in spring 2023 but more mites in summer 2023 than in summer 2022 (Fig. 2). These differences could not be explained by any field observations in the apiaries and their corresponding study areas. They may be due to the impact of unknown environmental factors affecting both the honey bees and the mites, including mite immigration, as well as genetic factors of the colonies.

As suggested by previous studies (Ritter and Leclercq 1987, Sakofski et al. 1990, Greatti et al. 1992), mite immigration occurred even at the beginning of the beekeeping season (Figs. 5 and 6). In our study, immigration accounted for 17–48% of the final colony infestation level (Fig. 6, Supplementary Material SM3). This proportion is also likely to have depended on the initial mite infestation level of the evaluated colonies. As there were no winter miticide treatments between consecutive years of our experiments, our colonies started with potentially more mites than colonies in selection programs where winter treatments are performed, as recommended in the frame of good beekeeping practice (Imdorf et al. 1996, Charrière and Imdorf 2002). In such programs, mite immigration during spring and early summer may have a higher impact on mite infestation level recorded in summer. It is also likely that, as the result of different levels of resistance behaviors, the number of offspring per mother mite and the number of reproductive cycles per mite would vary more between colonies when the latter are not all headed by sister queens, such as in our experiment. In our study, this particular design was required to be able to attribute the immigration estimated in the treated group to the control colonies. In more genetically diverse honey bee colonies, the efficacy of resistance behaviors in counterbalancing the impact of mite immigration may vary, depending on the colony considered: resistance traits could eliminate a varying proportion of the offspring of immigrated mites. In addition, at least to a small extent, mite immigration may also depend on genetic factors of the target colony or the immigrating mites, potentially providing even more room for variation in mite immigration between colonies located in the same apiary.

Based on our results, the impact of mite immigration on the capacity to clearly distinguish honey bee colonies according to their infestation levels, as required for selection, was moderate: depending on the year and season, mite infestation levels of single colonies could be significantly distinguished, irrespective of the impact of mite immigration, in 72–93% of pairwise comparisons (Fig. 7, Supplementary Material SM4). These proportions are relatively high. There are two possible reasons for this finding. First, between-colony genetic diversity could have been relatively high, even among colonies headed by sister queens, resulting in variable resistance behaviors. This would enable selection for resistance traits under beekeeping conditions. Second, despite our efforts to homogenize as much as possible the initial mite infestation levels within each apiary when creating the experimental colonies through bee packages, there have already been, according to the adult worker samples, an unneglectable variation of the initial mite infestation level among these colonies (Fig. 2). Such initial variation could have led to differences in final infestation levels, which could not be related to resistance traits or horizontal mite flows, therefore possibly partly blurring our conclusions. We however decided not to correct our final colony infestation levels due to the oxalic acid treatment for the initial infestation level assessed by the worker samples or natural mite fall. Indeed, the different methods used to measure infestation levels at different time points could reflect different colony traits, as highlighted by their low-to-medium pairwise correlations (Table 1).

We observed variations in the number of immigrating mites and their impact on final colony infestation levels depending on years and experimental apiaries. These variations were not related to regional differences in colony density or mite infestation level of the neighboring colonies, as mite infestation level, recorded in beekeepers’ colonies in spring and summer, did not differ significantly between the study areas (Table 3). This result indicates that the infestation levels of the neighboring colonies in the study areas could not be predicted based on local colony density. The proportion of beekeeper’s colonies whose infestation levels exceeded locally recommended thresholds varied between the study areas. However, this variation was not always reflected in mite immigration data retrieved from the experimental colonies. For example, in 2023, the areas with the highest proportions of highly infested colonies in summer (HE [29%], GH [25%], Fig. 8) corresponded to the experimental apiaries with the highest mite immigration (Fig. 6), while in 2022, the higher proportion measured in the MB area (29%) (Fig. 8) did not apparently lead to more mite immigration (Fig. 6). A significant study area*year interaction was observed (Table 3), due to lower infestation levels in the colonies in the MB study area in 2023. This finding could be explained by colony losses during winter 2022–2023 for beekeepers usually experiencing higher infestation levels in this study area (see the decrease in the number of sampled colonies between summer 2022 [115] and spring 2023 [89], Table 2), increasing the proportion of beekeepers with lower infestation levels due to better management.

The fact that the infestation of neighboring colonies could not directly be linked to mite immigration could mean that our experiment may not have identified all the factors leading to horizontal mite flows between colonies. First, not all colonies could be sampled (Table 2), as some beekeepers did not wish to participate in the study. Second, the frequency of sampling was likely not sufficient. It is possible that some highly infested colonies have been lost, euthanized, or moved out of the study area by the beekeepers between both samplings. However, more frequent sampling would probably have discouraged many beekeepers from participating in the study. Third, these results could also mean that mite immigration spans further than the 2 km radius of our study areas, depending on foraging distance. Given the number of colonies already included in the study areas, extending this radius would have been difficult, leading to a sharp increase in the number of neighboring colonies to sample: performing such investigations could not be routinely performed before setting up evaluation apiaries in selection programs. Fourth, unpredictable effects of a small, unknown fraction of the surveyed colonies, not necessarily the most infested, may have contributed to the majority of the observed mite flows, for instance because of specific foraging routes of a few colonies. Such events cannot be traced and could lead to the absence of a relationship between the regional infestation level and mite immigration targeting the experimental colonies. Fifth, multiple environmental effects other than colony density could have a strong, local influence on mite immigration. Examples of such effects are nectar and pollen resources, beekeeping management practices, including colony feeding, weather conditions, land use, and landscape connectivity (Lander et al. 2013). Understanding the single impacts of such environmental effects would require numerous repetitions of a similar experiment in regions with contrasting and precisely characterized environmental conditions. In either case, as the effect of the individual apiaries on colony infestation level was highly significant (Table 3), the results also indicate that more than the local colony density, the practice of each beekeeper is likely to have had the highest impact on the infestation levels of his/her colonies (Lerch 2020, Hernandez et al. 2022).

As horizontal mite flows are likely to be far higher in the fall (Ritter and Leclercq 1987, Sakofski et al. 1990, Büchler and Hoffmann 1991, Greatti et al. 1992), their impact on biases when evaluating mite infestation levels during the following season should be considered. In our study, we limited our analysis to the period between spring and summer, that is, the time window when most selection programs evaluate mite infestation levels of their colonies. Colonies for phenotypic evaluation are usually established in summer, to be headed by the recently produced new generation of queens, and traits are recorded the following year. Even if mite infestation level is standardized when colonies are established, late-season mite immigration may differentially affect the number of mites with which the colonies overwinter. Even applying a winter treatment of high and expected identical efficacy to all colonies may not be a solution to prevent biases. Despite such treatment, these colonies may start the following season with variable infestation levels, even if the levels are too low to be detected when colony evaluation starts in spring. This situation could also provide biased infestation phenotypes for selection. One solution for better standardization could be to perform a winter treatment and, later, to add an equal and relatively high number of mites to each colony. However, mites are usually difficult to obtain from donor colonies at the beginning of spring, as highly infested colonies usually do not survive winter.

More generally, the low correlations between-colony infestation levels obtained by different methods (Table 1) complicate selection by making the choice of the right trait unclear. We expect mite mortality counts following the final oxalic acid treatment to best reflect the actual colony infestation level in summer. This evaluation method could perhaps be integrated into selection programs. The sum of natural mite fall, which correlated best with the final colony infestation level, could be a suitable alternative but is much more work intensive, as it requires monitoring mite mortality during several months.

In conclusion, the present study indicates that mite immigration can take place in spring and early summer. Depending on the initial infestation level of the considered colonies, mite immigration could have a more or less strong impact on the infestation levels used for selection. Under the conditions in the present study, we detected no clear effect of the density of neighboring colonies or of their infestation levels on mite immigration. As a result, we are not able to provide a threshold to beekeepers regarding the maximal local colony density when setting up an apiary for selecting less infested colonies. We consider that colony evaluation may be continued as currently performed. Future studies could transpose our methods to other regions to verify whether our conclusion could be generalized, or to determine whether regions with even lower colony densities—rare in Switzerland—could be more appropriate in other countries. Mite infestation dynamics are clearly influenced by multiple, complex environmental factors, which limits reliable estimations of heritabilities and hinders fast selection progresses on the long-term road toward *V. destructor*-resistant colonies. In the short term, from a beekeeping perspective, the highly significant effects of the individual apiaries on mite infestation levels highlight the importance of apiary location choice and/or mite management strategy as effective action levers to keep mite infestation level under control and honey bee colonies healthy.

## Supplementary Material

ieae068_suppl_Supplementary_Material_S1

ieae068_suppl_Supplementary_Material_S2

ieae068_suppl_Supplementary_Material_S3

ieae068_suppl_Supplementary_Material_S4

ieae068_suppl_Supplementary_Material_S5

ieae068_suppl_Supplementary_Data
