# Supplementary material for: Do Varroa destructor (Acari: Varroidae) mite flows between Apis mellifera (Hymenoptera: Apidae) colonies bias colony infestation evaluation for resistance selection?
Source: J Insect Sci. 2024 Jul 11;24(4):3. doi: 10.1093/jisesa/ieae068 (PMC11237995; doi:10.1093/jisesa/ieae068)
Supplement: ieae068_suppl_Supplementary_Material_S1 [file ieae068_suppl_supplementary_material_s1.pptx]

## Slide 1
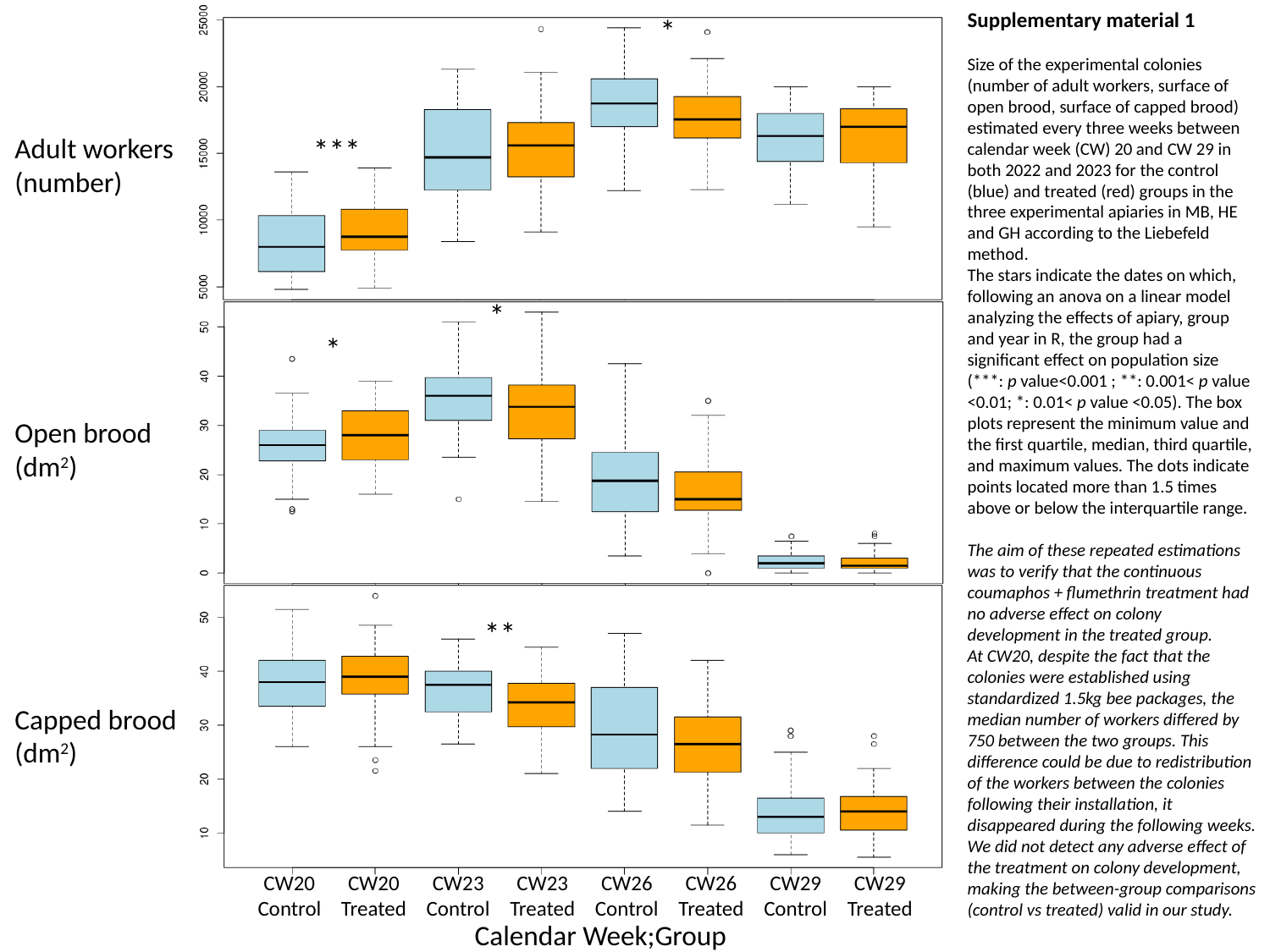

Supplementary material 1
Size of the experimental colonies (number of adult workers, surface of open brood, surface of capped brood) estimated every three weeks between calendar week (CW) 20 and CW 29 in both 2022 and 2023 for the control (blue) and treated (red) groups in the three experimental apiaries in MB, HE and GH according to the Liebefeld method.
The stars indicate the dates on which, following an anova on a linear model analyzing the effects of apiary, group and year in R, the group had a significant effect on population size (***: p value<0.001 ; **: 0.001< p value <0.01; *: 0.01< p value <0.05). The box plots represent the minimum value and the first quartile, median, third quartile, and maximum values. The dots indicate points located more than 1.5 times above or below the interquartile range.
The aim of these repeated estimations was to verify that the continuous coumaphos + flumethrin treatment had no adverse effect on colony development in the treated group.
At CW20, despite the fact that the colonies were established using standardized 1.5kg bee packages, the median number of workers differed by 750 between the two groups. This difference could be due to redistribution of the workers between the colonies following their installation, it disappeared during the following weeks. We did not detect any adverse effect of the treatment on colony development, making the between-group comparisons (control vs treated) valid in our study.
*
***
Adult workers
(number)
*
*
Open brood
(dm2)
**
Capped brood
(dm2)
CW20
Control
CW20
Treated
CW23
Control
CW23
Treated
CW26
Control
CW26
Treated
CW29
Control
CW29
Treated
Calendar Week;Group
