# Supplementary material for: Do Varroa destructor (Acari: Varroidae) mite flows between Apis mellifera (Hymenoptera: Apidae) colonies bias colony infestation evaluation for resistance selection?
Source: J Insect Sci. 2024 Jul 11;24(4):3. doi: 10.1093/jisesa/ieae068 (PMC11237995; doi:10.1093/jisesa/ieae068)
Supplement: ieae068_suppl_Supplementary_Material_S2 [file ieae068_suppl_supplementary_material_s2.pptx]

## Slide 1
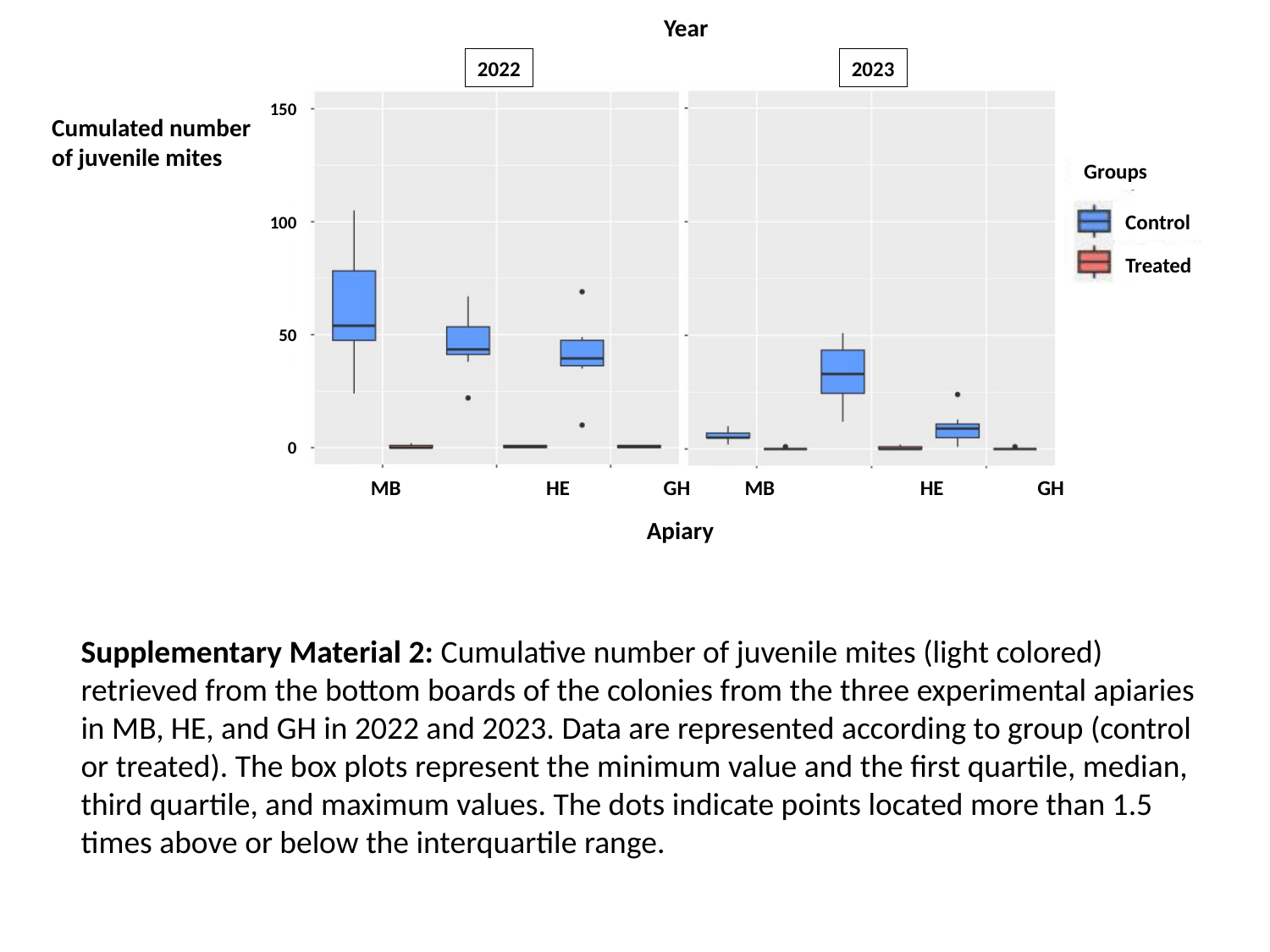

Year
2022
2023
150
Cumulated number of juvenile mites
Groups
Control
100
Treated
50
0
MB	 HE	 GH
MB	 HE	 GH
Apiary
Supplementary Material 2: Cumulative number of juvenile mites (light colored) retrieved from the bottom boards of the colonies from the three experimental apiaries in MB, HE, and GH in 2022 and 2023. Data are represented according to group (control or treated). The box plots represent the minimum value and the first quartile, median, third quartile, and maximum values. The dots indicate points located more than 1.5 times above or below the interquartile range.
