# Supplementary material for: Do Varroa destructor (Acari: Varroidae) mite flows between Apis mellifera (Hymenoptera: Apidae) colonies bias colony infestation evaluation for resistance selection?
Source: J Insect Sci. 2024 Jul 11;24(4):3. doi: 10.1093/jisesa/ieae068 (PMC11237995; doi:10.1093/jisesa/ieae068)
Supplement: ieae068_suppl_Supplementary_Material_S3 [file ieae068_suppl_supplementary_material_s3.docx]

**Supplementary material 3:** Mean (μ) and standard deviation (σ) of the final colony infestation level and mean (μ) and standard deviation (σ) of the estimated impact of mite immigration and proportion of mites in the colony at the time of the final oxalic acid treatment that could be explained by mite immigration in 2022 and 2023. The number of colonies for which complete datasets were obtained (N) is also indicated.

| Year | Apiary | μ (σ) final mite infestation  (control group) | μ (σ) estimated impact of mite immigration (treated group) | Percentage of the mites of control group potentially explained by immigration  (as estimated by the impact in the treated group) |
| --- | --- | --- | --- | --- |
| 2022 | MB | 887 (216) ; N=10 | 152 (41) ; N=10 | 17% |
|  | HE | 723 (170) ; N=10 | 166 (37) ; N=10 | 23% |
|  | GH | 771 (201); N=10 | 136 (36) ; N=10 | 18% |
| 2023 | MB | 255 (90) ; N=9 | 123 (35) ; N=9 | 48% |
|  | HE | 1159 (423) ; N= 10 | 306 (59) ; N=10 | 26% |
|  | GH | 617 (228) ; N=9 | 297 (94) ; N=10 | 48% |
