# Supplementary material for: Do Varroa destructor (Acari: Varroidae) mite flows between Apis mellifera (Hymenoptera: Apidae) colonies bias colony infestation evaluation for resistance selection?
Source: J Insect Sci. 2024 Jul 11;24(4):3. doi: 10.1093/jisesa/ieae068 (PMC11237995; doi:10.1093/jisesa/ieae068)
Supplement: ieae068_suppl_Supplementary_Material_S4 [file ieae068_suppl_supplementary_material_s4.docx]

**Supplementary material 4:** Impact of mite immigration on colony discrimination according to their final infestation level for 2022 and 2023. The results of the Kruskal–Wallis rank-sum tests and the proportion of significant differences between colonies for their estimated unbiased final infestation level following pairwise Wilcoxon rank-sum tests are provided. The *p*-values of the Wilcoxon rank-sum tests were adjusted according to the Benjamini–Hochberg (BH) procedure.

| Year | Apiary | Significant effect of colony on infestation? Results of Kruskal-Wallis rank sum test | Number of treated colonies to estimate the impact of mite immigration | Number of control colonies | Number of pairwise comparisons between infestations of control colonies corrected for the estimated impact of mite immigration retrieved from the treated colonies | Number of pairwise comparisons leading to significant (p<0.05) differences between colonies following Wilcoxon rank sum tests (p adjustment method: BH) | % of pairwise comparisons with significant differences between colonies |
| --- | --- | --- | --- | --- | --- | --- | --- |
| 2022 | MB | χ^2^= 93.4,  df = 9,  p = 3.4e-16 | 10 | 10 | 45 | 42 | 93% |
|  | HE | χ^2^= 92.7,  df = 9,  p = 4.6e-16 | 10 | 10 | 45 | 42 | 93% |
|  | GH | χ^2^= 91.5,  df = 9,  p = 8.2e-16 | 10 | 10 | 45 | 41 | 91% |
| 2023 | MB | χ^2^= 69.0,  df = 8,  p = 7.8e-12 | 9 | 9 | 36 | 26 | 72% |
|  | HE | χ^2^= 92.1,  df = 9,  p = 6.1e-16 | 10 | 10 | 45 | 41 | 91% |
|  | GH | χ^2^= 75.9,  df = 8,  p = 3.3e-13 | 10 | 9 | 36 | 29 | 81% |
