# Supplementary material for: Do Varroa destructor (Acari: Varroidae) mite flows between Apis mellifera (Hymenoptera: Apidae) colonies bias colony infestation evaluation for resistance selection?
Source: J Insect Sci. 2024 Jul 11;24(4):3. doi: 10.1093/jisesa/ieae068 (PMC11237995; doi:10.1093/jisesa/ieae068)
Supplement: ieae068_suppl_Supplementary_Material_S5 [file ieae068_suppl_supplementary_material_s5.pptx]

## Slide 1
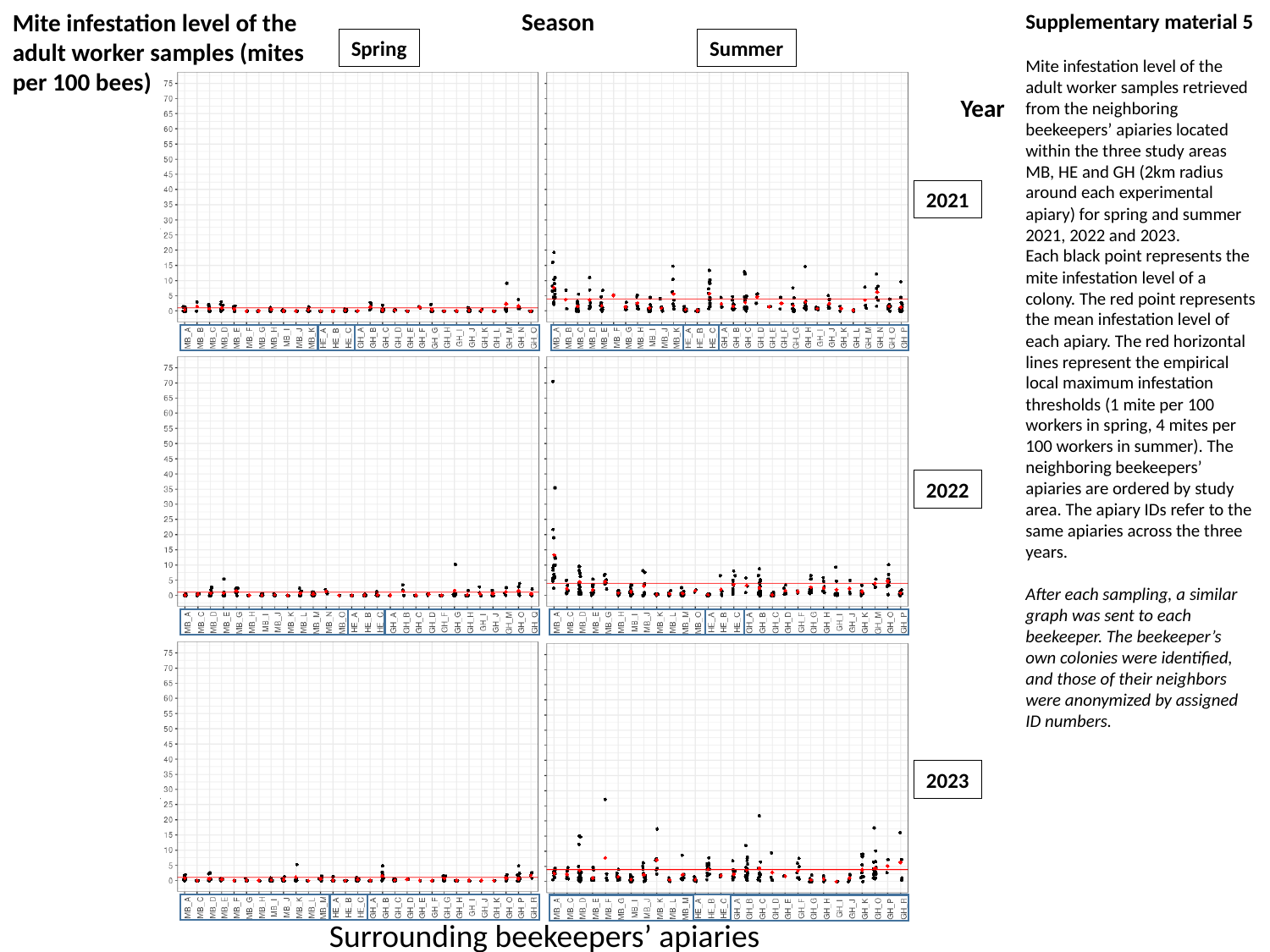

Season
Mite infestation level of the adult worker samples (mites per 100 bees)
Supplementary material 5
Mite infestation level of the adult worker samples retrieved from the neighboring beekeepers’ apiaries located within the three study areas MB, HE and GH (2km radius around each experimental apiary) for spring and summer 2021, 2022 and 2023.
Each black point represents the mite infestation level of a colony. The red point represents the mean infestation level of each apiary. The red horizontal lines represent the empirical local maximum infestation thresholds (1 mite per 100 workers in spring, 4 mites per 100 workers in summer). The neighboring beekeepers’ apiaries are ordered by study area. The apiary IDs refer to the same apiaries across the three years.
After each sampling, a similar graph was sent to each beekeeper. The beekeeper’s own colonies were identified, and those of their neighbors were anonymized by assigned ID numbers.
Summer
Spring
Year
2021
2022
2023
Surrounding beekeepers’ apiaries
