# Supplementary material for: Do Varroa destructor (Acari: Varroidae) mite flows between Apis mellifera (Hymenoptera: Apidae) colonies bias colony infestation evaluation for resistance selection?
Source: J Insect Sci. 2024 Jul 11;24(4):3. doi: 10.1093/jisesa/ieae068 (PMC11237995; doi:10.1093/jisesa/ieae068)
Supplement: ieae068_suppl_Supplementary_Data [file ieae068_suppl_supplementary_data.docx]

**Supplementary material (SM)**

**SM1.** Size of the experimental colonies (number of adult workers, surface of open brood, surface of capped brood) estimated every three weeks between calendar week (CW) 20 and CW 29 in both 2022 and 2023 for the control (blue) and treated (red) groups in the three experimental apiaries in MB, HE, and GH according to the Liebefeld method. The stars indicate the dates on which, following an ANOVA on a linear model analyzing the effects of apiary, group and year in R, the group had a significant effect on population size (***: *p* value < 0.001 ; **: 0.001 < *p* value < 0.01; *: 0.01 < *p* value < 0.05). The box plots represent the minimum value and the first quartile, median, third quartile, and maximum values. The dots indicate points located more than 1.5 times above or below the interquartile range. The aim of these repeated estimations was to verify that continuous coumaphos + flumethrin treatment had no adverse effect on colony development in the treated group. At CW 20, despite the fact that the colonies were established using standardized 1.5 kg bee packages, the median number of workers differed by 750 between the two groups. This difference could be due to redistribution of the workers between the colonies following their installation, it disappeared during the following weeks. We did not detect any adverse effect of the treatment on colony development, making the between-group comparisons (control vs treated) valid in our study.

**SM2.** Cumulative number of juvenile mites (light colored) retrieved from the bottom boards of the colonies from the three experimental apiaries in MB, HE, and GH in 2022 and 2023. Data are represented according to group (control or treated). The box plots represent the minimum value and the first quartile, median, third quartile, and maximum values. The dots indicate points located more than 1.5 times above or below the interquartile range.

**SM3.** Mean (μ) and standard deviation (σ) of the final colony infestation level and mean (μ) and standard deviation (σ) of the estimated impact of mite immigration and proportion of mites in the colony at the time of the final oxalic acid treatment that could be explained by mite immigration in 2022 and 2023. The number of colonies for which complete datasets were obtained (N) is also indicated.

**SM4.** Impact of mite immigration on colony discrimination according to their final infestation level for 2022 and 2023. The results of the Kruskal–Wallis rank-sum tests and the proportion of significant differences between colonies for their estimated unbiased final infestation level following pairwise Wilcoxon rank-sum tests are provided. The *p*-values of the Wilcoxon rank-sum tests were adjusted according to the Benjamini–Hochberg (BH) procedure.

**SM5.**Mite infestation level of the adult worker samples retrieved from the beekeepers’ apiaries located within the three study areas MB, HE, and GH (2km radius around each experimental apiary) for spring and summer 2021, 2022, and 2023. Each black point represents the mite infestation level of a colony. The red point represents the mean infestation level of each apiary. The red horizontal lines represent the empirical local maximum infestation thresholds (1 mite per 100 workers in spring, 4 mites per 100 workers in summer). The neighboring beekeepers’ apiaries are ordered by study area. The apiary IDs refer to the same apiaries across the three years. After each sampling, a similar graph was sent to each beekeeper. The beekeeper’s own colonies were identified, and those of their neighbors were anonymized by assigned ID numbers.
